# Using Neural Networks to Generate Inferential Roles for Natural Language

**DOI:** 10.3389/fpsyg.2017.02335

**Published:** 2018-01-17

**Authors:** Peter Blouw, Chris Eliasmith

**Affiliations:** Center for Theoretical Neuroscience, University of Waterloo, Waterloo, ON, Canada

**Keywords:** natural language inference, recursive neural networks, language comprehension, semantics

## Abstract

Neural networks have long been used to study linguistic phenomena spanning the domains of phonology, morphology, syntax, and semantics. Of these domains, semantics is somewhat unique in that there is little clarity concerning what a model needs to be able to do in order to provide an account of how the meanings of complex linguistic expressions, such as sentences, are understood. We argue that one thing such models need to be able to do is generate predictions about which further sentences are likely to follow from a given sentence; these define the sentence's “inferential role.” We then show that it is possible to train a tree-structured neural network model to generate very simple examples of such inferential roles using the recently released Stanford Natural Language Inference (SNLI) dataset. On an empirical front, we evaluate the performance of this model by reporting entailment prediction accuracies on a set of test sentences not present in the training data. We also report the results of a simple study that compares human plausibility ratings for both human-generated and model-generated entailments for a random selection of sentences in this test set. On a more theoretical front, we argue in favor of a revision to some common assumptions about semantics: understanding a linguistic expression is not only a matter of mapping it onto a representation that somehow constitutes its meaning; rather, understanding a linguistic expression is mainly a matter of being able to draw certain inferences. Inference should accordingly be at the core of any model of semantic cognition.

## 1. Introduction

By most accounts, linguistic comprehension is the result of cognitive processes that map between sounds and mental representations of meaning (e.g., Smolensky and Legendre, 2006; Pickering and Garrod, 2013; Christiansen and Chater, 2016). An obvious challenge for these accounts is to provide a good theoretical characterization of the relevant representations. Numerous proposals can be found in the literature, but there is no obvious consensus regarding their relative merits.

Arguably, the reason for this lack of consensus is that linguistic comprehension is itself a somewhat vague and ill-defined phenomenon. In the context of efforts to *model* linguistic comprehension, for instance, it is not entirely obvious what a model needs to be able to do in order to provide an account of how people understand complex linguistic expressions such as phrases and sentences.

In this paper, we argue that one thing models of linguistic comprehension need to be able to do is generate predictions about what follows from a given sentence during a conversation. For example, to understand the statement “The dancers parade down the street,” one must be able recognize that the dancers are outside, that they are not standing still, and that there is likely a surrounding audience, along with various other things. Comprehending a sentence therefore involves drawing inferences that identify the expected consequences of the occurrence of the sentence in the linguistic environment. And since comprehending a sentence involves comprehending its *meaning*, it follows that the meaning of an expression is at least partly determined by the inferences it licenses (Sellars, 1953; Brandom, 1994, 2000). The collections of inferences licensed by a particular sentence, in turn, constitutes its inferential role (Brandom, 1994).

Our approach can be thought of as an extension of two important trends in previous research. On a technical front, the explanatory successes of probabilistic and neural network models in psycholinguistics have motivated the view that language learning is a kind of skill acquisition, wherein a learner develops the ability to *process and use* linguistic expressions correctly (Elman, 1990, 1991; Seidenberg, 1997; Tomasello, 2003; Chater and Manning, 2006; Christiansen and Chater, 2016). To explain with an example, an artificial neural network learns a set of parameters (i.e., connection weights) that approximate a function defined by a set of input-output pairs. These pairs might map words to collections of phonemes during a generation task, or to collections of property concepts during an interpretation task (see e.g., McClelland et al., 2010). Our work extends this research to account for more sophisticated linguistic phenomena that involve inferences defined with respect to complete sentences (cf. St. John and McClelland, 1990; Rabovsky et al., in review).

On a more theoretical front, a considerable amount of philosophical research has been directed toward explaining the significance that attributions of “understanding” have for semantic theory (Dennett, 1987, 1991; Brandom, 1994, 2000). One lesson to draw from this prior work is that the meaning of a linguistic expression is something that determines what a person who understands the expression is likely to say and do in various situations (Blouw, 2017). Or put another way, meanings can be thought of as codifying implicit expectations that people have regarding certain effects of language use. Our work builds on these philosophical insights by working toward a formal characterization of the role that linguistic expressions play in licensing certain predictions when one adopts what Dennett (1987, 1991) refers to as the “intentional stance.” To explain, adopting the intentional stance involves making predictions about a system's behavior using linguistically specified mental states attributions, such as “*X* understands *Y*,” where is *X* is a system and *Y* is a sentence in a natural language. So, to return to an earlier example, questions about the meaning of a sentence like “The dancers parade down the street” can be reformulated as questions about the predictions and inferences that are licensed by the attribution of intentional states involving this sentence. More specifically, attributions of understanding license the prediction that certain questions (e.g., “Where are the dancers?”) get responded to with certain answers (e.g., “They are outside”)^1^.

**Figure 1:**
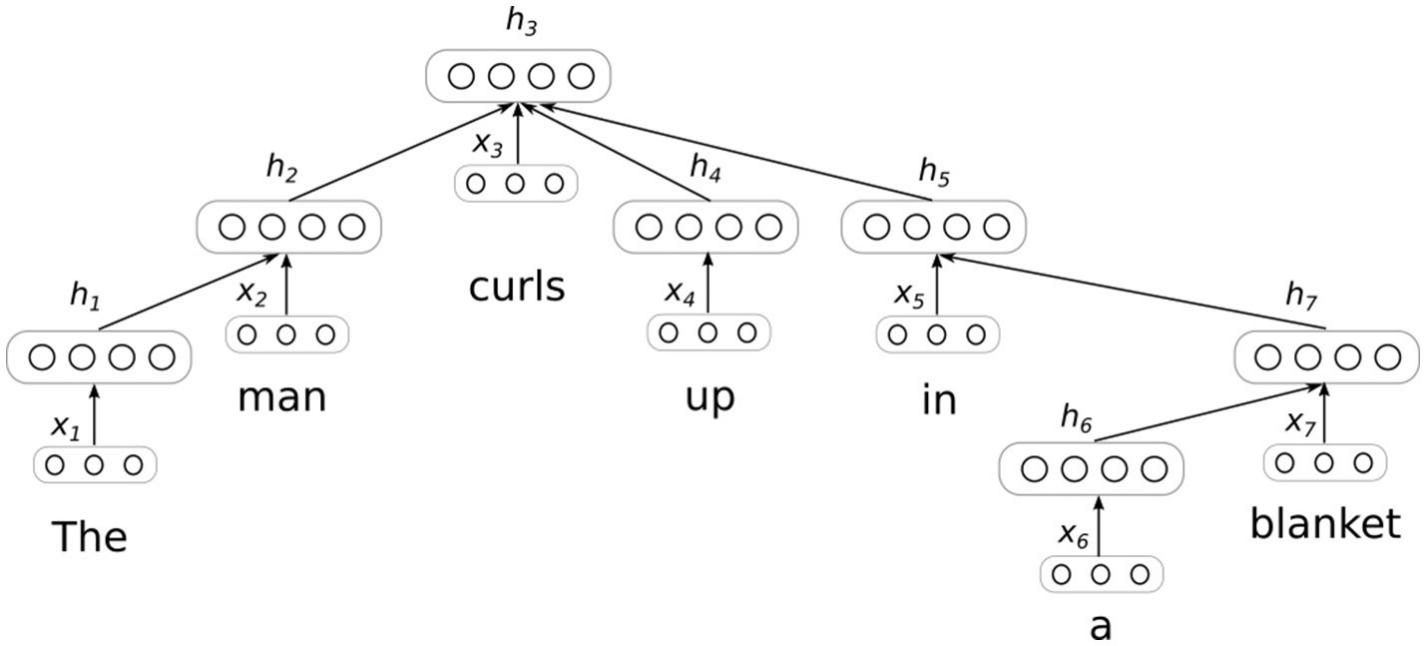
Sentence encoding with a dependency tree recursive neural network (DT-RNN). A dependency parser is used to produce the computational graph for a neural network, which is then used to produce a distributed representation of sentence by merging distributed representations of individual words. The layers marked with *x* correspond to input word embeddings, while layers marked with *h* correspond to the tree's encoding of these words. Figure adapted from Socher et al. (2014).

To work toward formalizing these aspects of intentional interpretation, we introduce a neural network model that learns to generate sentences that are the inferential consequences of its inputs. The model functions by first encoding a sentence into a distributed representation, and then decoding this representation to produce a new sentence. The encoding procedure involves dynamically generating a tree-structured network layout of the sort depicted in Figure 1. Once a sentence encoding is produced using this network, it is fed through an “inverse” tree-structured network to produce a predicted sentence. Interestingly, different inverse or decoding networks can be used to generate different sentences from a single encoding. To train the model parameters (i.e., the network weights shared across different tree structures) we use the Stanford Natural Language Inference (SNLI) dataset (Bowman et al., 2015). The goal of the model is to characterize a very basic portion of the predictions that are licensed by uses of the sentences it is provided as input. Currently, the model's predictions tend to favor the generation of sentences with roughly the same meanings as its inputs, but it is also able to generate more interesting predictions of the sort that are a necessary precondition for linguistic comprehension.

In what follows, we first describe the model and then empirically evaluate its ability to produce plausible entailments for sentences unseen in the training data. We present experimentally produced plausibility ratings for a random collection of generated sentences, and from these ratings conclude that the model captures something important about the inferential relations amongst ordinary linguistic expressions. We then perform a number of further analyses that illustrate how the model is able generalize by “interpolating” between familiar examples of inferential transitions. Finally, we discuss the implications of this work for both the study of semantics and the study of cognition more generally.

## 2. Methods

### 2.1. Tree-structured neural networks

To build our model, we take advantage of recently developed techniques for using neural networks to define composition functions that merge distributed representations of words into distributed representations of phrases and sentences (Socher et al., 2012, 2014). The core idea behind these techniques is to produce a parse tree for a sentence, and then transform the tree into a neural network by replacing its edges with weights and its nodes with layers of artificial neurons. Activation is then propagated up the tree by providing input to layers that correspond to certain nodes, as shown in Figure 1. The input at each node is typically a distributed representation or “embedding” corresponding to a single word (see e.g., Landauer and Dumais, 1997; Jones and Mewhort, 2007; Turney and Pantel, 2010; Mikolov et al., 2013).

It is possible to apply these methods using arbitrary tree structures, and we adopt a dependency-based syntax in the experiments described below. There are three reasons for this choice (Socher et al., 2014). First, the assignment of different network weights to different dependency relations allows for the creation of networks that are more sensitive to syntactic information. Second, the semantic role of an individual word can often be read off of the dependency relation it bears to a head word, which allows for the creation of networks that are also sensitive to semantic information. Finally, dependency trees are less sensitive to arbitrary differences in word order, which helps to ensure that simple variations on a sentence get mapped to similar distributed representations. The specific model we adapt—the dependency-based tree-structured neural network (DT-RNN)—is introduced in Socher et al. (2014).

Some formal details concerning the behavior of DT-RNNs are helpful at this point. First, an input sentence *s* is converted into a list of pairs, such that *s* = [(*w*_1_, *x*_1_), (*w*_2_, *x*_2_), …(*w*_*n*_, *x*_*n*_)], where *w* is a word and *x* is the corresponding word embedding (i.e., a distributed representation produced using word2vec). Next, a dependency parser is used to produce a tree that orders the words in the sentence in terms of parent-child relations. Each node in this tree is then assigned an embedding in a two-step manner. First, all of the leaf nodes in the tree (i.e., nodes that do not depend on other nodes) are assigned embeddings by applying a simple transformation to their underlying word embeddings:

(1) $$\begin{array}{r} h_{i}=f\left(W_{v}x_{i}+b\right) \end{array}$$

where *h*_*i*_ is the embedding for some leaf node *i* in the tree, *x*_*i*_ is the embedding for the word corresponding to this node, *W*_*v*_ is a matrix that transforms word representations, *b* is a bias term, and *f* is an element-wise nonlinearity. Second, embeddings are recursively assigned to all of the non-leaf nodes by composing the embeddings of their children as follows:

(2) $$\begin{array}{r} h_{i}=f\left(W_{v}x_{i}+\underset{j\in C\left(i\right)}{\sum }W_{R\left(i,j\right)}\cdot h_{j}+b\right) \end{array}$$

where *h*_*i*_ is again the embedding for some node *i* in the tree, *x*_*i*_ is the embedding for the word corresponding to this node, *j* is an index that ranges over the children, *C*(*i*), of the node *i*, and *W*_*R*(*i,j*)_ is a matrix associated with the specific dependency relation between node *i* and its *j*th child. *h*_*j*_ is the embedding corresponding to this child. So, in the example tree in Figure 1, the embeddings for nodes 1, 4, and 6 would be computed first, since these nodes have no children. Then, embeddings will be computed for any nodes whose children now all have assigned embeddings (in this case, nodes 2 and 7). And so on, until an embedding is computed for every node.

Model training is done via backpropagation through structure (Goller and Kuchler, 1996) and requires that a cost function be defined for the sentence embeddings produced at the root of each tree. The free parameters are the weights *W*_*v*_ and *W*_*r*∈*R*_, along with the bias term *b*. Word embeddings can also be fine-tuned over the course of training. The number of dependency relations, and hence the number of weight matrices in the model, depends on the specific syntactic formalism that is used. In the experiments described below, a standard set of 45 dependency relations defines the syntax that is used by the model's parser.

### 2.2. Cost functions for entailment generation

Choosing an appropriate cost function for a recursive neural network can be difficult, since it is not always clear what makes for a “good” sentence embedding. It is accordingly common to see these networks applied to narrow classification tasks such as the prediction of sentiment ratings (e.g., Socher et al., 2012). Our goal is define an optimization objective that accounts for the principle that understanding a linguistic expression involves drawing inferences about what follows from it.

To accomplish this goal, we define a model composed of two DT-RNNs, one that encodes an input sentence into a distributed representation, and another that decodes this representation into a new sentence that is entailed by the input sentence. This model is inspired by Iyyer et al.'s (2014) work using DT-RNNs analogously to autoencoders, but introduces a decoding procedure that computes an appropriate response to the input sentence, rather than merely reconstructing it. It is then possible to iterate these encoding and decoding procedures to produce chains of entailments, as proposed by Kolesnyk et al. (2016), who use a sequence-based encoder and decoder. It is also possible analyze the effect on the decoding procedure of substituting individual words and phrases into an input sentence, as shown in section 3.4 below.

**Figure 2:**
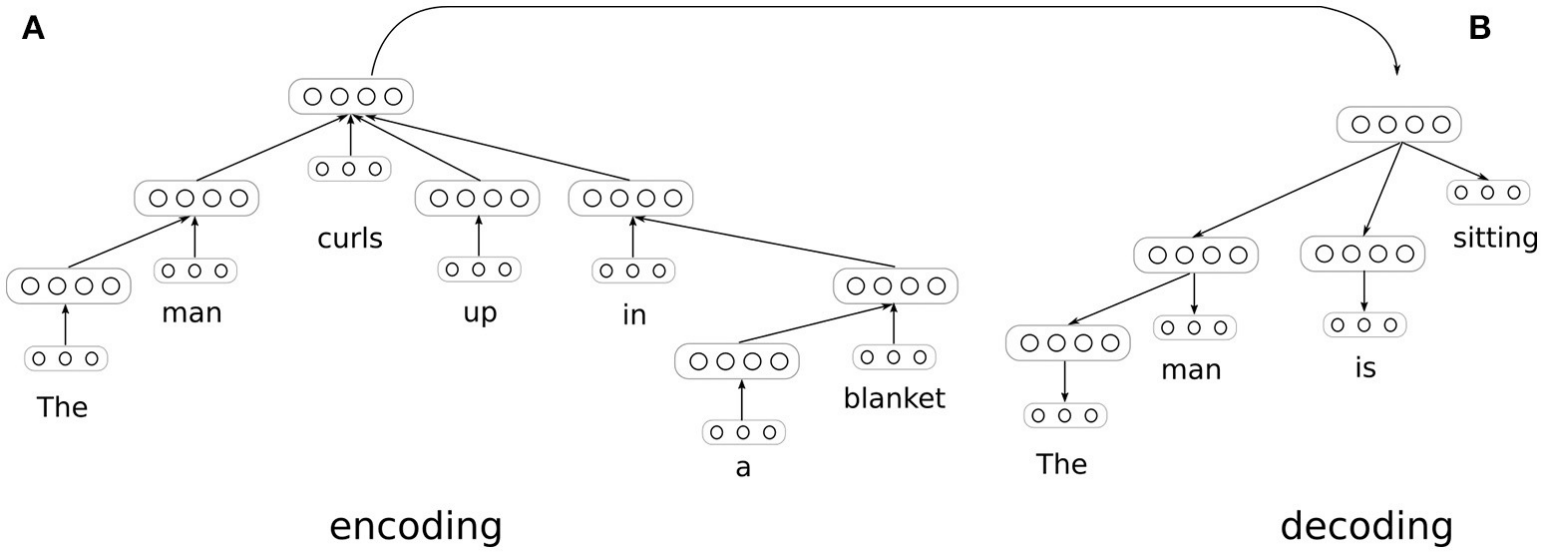
Generating entailments with paired encoder and decoder DT-RNNs. The decoder network computes a probability distribution over words at each node, conditioned on the sentence representation produced by the encoder. The parameters of both the encoder and decoder are trained via backpropagation through structure using error derivatives supplied at each node in the decoding tree. The encoder and decoder trees are dynamically generated for each pair of sentences in the training data.

The model is trained on pairs of sentences standing in entailment relations. A dependency parser^2^ is again used to produce a tree-structured network for each sentence, but the network associated with the second sentence is run in reverse, as shown in Figure 2. A word prediction is generated at each node in this second tree using a softmax classifier, which allows us to define a cross-entropy loss function over nodes and trees as follows:

(3) $$\begin{array}{r} J\left(\theta \right)=-\underset{i}{\sum }\underset{j}{\sum }t_{j}^{\left(i\right)}\log p\left(c_{j}^{\left(i\right)}|s_{i}\right) \end{array}$$

where $t_{j}^{\left(i\right)}$ is the target probability (i.e., 1) for the correct word at the *j*th node in the *i*th training example, $p\left(c_{j}^{\left(i\right)}|s_{i}\right)$ is the computed probability for this word given the input sentence *s*_*i*_, and θ is the set of combined parameters for the encoder and decoder DT-RNNs. Intuitively, this cost function penalizes model parameters that fail to assign a high joint probability to the collection of word predictions in the decoder that correspond to the correct entailment for a given input sentence. More formally, the training objective is to maximize the log probability of the example entailments provided in the training data.

Learning is done via stochastic gradient descent by backpropogating through both the decoder and encoder tree for each training example. The result of training is a set of weights associated with dependencies for both encoding and decoding, a set of weights for predicting a distribution over words from a node embedding for each dependency, a set of biases (we allow dependency-specific biases), the input transformation matrix *W*_*v*_, and the softmax classifier weights. When the trained model is used to perform inference using a novel input sentence, the encoder DT-RNN is assembled into a tree using the learned encoding weights. The decoder DT-RNN is then also assembled into a tree using the learned decoding weights, and activation is propagated through the encoder and into the decoder to produce a probability distribution over words at each tree node. The words with the highest probability at each node are then used to construct the predicted entailment for the input sentence. The tree structure for the decoder can either be selected randomly or stipulated ahead of time.

### 2.3. Training data and training procedure

To train the encoder and decoder components of the model, we use a subset of the SNLI corpus (Bowman et al., 2015). This corpus is a recently released dataset consisting of 570,152 sentences pairs labeled with inferential relationships. The first sentence in each pair is referred to as the *premise*, while the second sentence is referred to as the *hypothesis*. If the hypothesis follows from the premise, then the pair is labeled as an example of entailment. If the hypothesis is inconsistent with the premise, the pair is labeled as an example of contradiction. And if the hypothesis might or might not be true given the premise, then the pair is labeled as neutral.

Each sentence pair is generated by providing a human annotator^3^ with an image caption (but not the corresponding image), and then asking them to write three further captions: one which is definitely also true of the image, one which might be true of the image, and one which is definitely not true of the image. To illustrate with an example, one initial caption is “Under a blue sky with white clouds, a child reaches up to touch the propeller of a plane standing parked on a field of grass,” and the annotator produced the following three additional captions: “A child is reaching to touch the propeller of a plane” (entailment), “A child is reaching to touch the propeller out of curiosity” (neutral), and “A child is playing with a ball” (contradiction). The use of image captions is designed to eliminate ambiguities concerning event and entity co-reference across the sentences in a given pair. Approximately ten percent of the resulting pairs were subject to a further validation step in which four additional annotators assigned them one of the three relationship labels. The results of this data validation suggest that inter-annotator agreement is very high, with ~98% of validated sentence pairs receiving a consensus label (i.e., at least three of the five annotators are in agreement).

Since our interest is in generating entailments, we only consider pairs labeled with the entailment relation. To reduce the amount of noise and complexity in the dataset, we also perform some simple pre-processing. First, we screen for misspelled words,^4^ and eliminate all sentence pairs containing a misspelling. The resulting vocabulary for the model consists of 22,495 words. Second, we eliminate all sentence pairs containing a sentence longer than 15 words in order to avoid fitting model parameters to a small number of very long sentences that produce highly complex dependency trees. After preprocessing, the data consists of a 106,246-pair training set, a 1,700-pair development set, and 1,666-pair test set. Within the training set, 89,458 premise sentences occur in a single training pair, while a further 3,998 sentences occur in multiple training pairs. The maximum number of pairs a unique premise sentence occurs in is 11 (i.e., their are 11 pairs in the training set with the same premise sentence), while the average number of pairs a premise sentence occurs in is 1.14. These statistics indicate that the model generally only has access to a single example of a correct inference for each premise sentence in the training data.

Model training is in accordance with the procedure described in the previous subsection. Specifically, for each pair of sentences in the training data, activation is propagated through the dynamically assembled encoder and decoder networks, so as to produce a probability distribution over words at each node in the decoder. An error signal determined by the difference between this computed distribution and the target distribution at each node is then used to compute a gradient for all of the parameters in the model, which include: (1) word2vec embeddings for each vocabulary item; (2) an encoding weight matrix, a decoding weight matrix, and bias vector for each of the 45 syntactic dependencies used by the SpaCy parser; (3) the embedding transformation matrix *W*_*v*_; and (4) softmax classifier weights for predicting words at nodes in the decoder. Prior to training, each set of weights associated with a syntactic dependency is initialized as a 300 × 300 identity matrix with mean-zero Gaussian noise for both the encoder and decoder. The word transformation matrix, *W*_*v*_, is initialized in the same way. Biases are initialized as the zero vector. Classifier weights are initialized using word2vec embeddings. Hyper-parameters include the learning rate, the annealing schedule, and the number of training iterations. These parameters were minimally hand-tuned by using the measure of entailment accuracy (described in the next section) on the development set. After tuning, the initial learning rate was set to 6e-4, and then progressively halved upon processing 45, 60, and 80 random samples of 10,000 pairs of items from the training set. Training was terminated after processing 100 samples of 10,000 pairs (i.e., roughly 10 passes through the training data).

As an initial illustration of the kind of model performance this training results in, Table 1 provides some examples of entailments produced for sentences drawn from the SNLI test set. The same decoding tree is used to produce each of these entailments, which suggests that the model is capable of producing plausible entailments under fixed syntactic constraints. It is also worth noting that each example here is only the *most probable* entailment given the decoding tree. It is therefore theoretically possible to compute ranked collections of entailments with each tree. To provide an illustration, Table 2 indicates how word probabilities are assigned to each node in a fixed decoding tree for a training sentence. As this example shows, the model learns probabilistic relations that allow it to go somewhat beyond the explicit meaning of the familiar input sentence. For instance, given that officers are mentioned in the input sentence, the model assigns a high probability to them being uniformed, blue (i.e., police), or green (i.e., military). Likewise, given that the officers are in a golf cart, the model assigns a high probability to them being on a course, or being with equipment (abbreviated as “equip” in Table 2). Given that the input sentence is only paired with the entailment “Two people in a golf cart” in the training data, these probabilistic relations indicate that model is learning to meaningfully generalize between example inferential transitions to some degree.^5^

**Table 1:** Examples of entailments generated from novel test sentences.

**Input sentence**	**Generated entailment**
The 3 dogs are cruising down the street.	The dogs are on the street.
Woman reading a book with a grocery tote.	A woman reading with a book.
The man in colorful shorts is barefoot.	The man wearing in the shorts.
A man laughing while at a restaurant.	A man laughing at a restaurant.
Two individuals are using a photo kiosk.	The people are at a kiosk.
A man pulling items on a cart.	A man pulling on a cart.
Three people are riding a carriage pulled by four horses.	A horses riding with a carriage.

**Table 2:** Decoder word probabilities for the sentence “Two officers sitting in a golf cart.”

the	0.89	uniformed	0.18	and	1.0	blue	0.16	people	0.27	are	0.23	in	0.43	cart	0.68	with	0.6	a	0.89	course	0.34
a	0.09	blue	0.15	or	0.0	green	0.12	officers	0.24	sitting	0.14	on	0.22	course	0.12	of	0.28	the	0.11	golf	0.19
some	0.01	old	0.07	of	0.0	yellow	0.11	men	0.14	people	0.03	with	0.18	equip.	0.04	in	0.08	an	0.01	equip.	0.18
an	0.0	green	0.03	but	0.0	white	0.05	they	0.04	sit	0.02	near	0.06	golf	0.03	on	0.03	this	0.0	cart	0.15
these	0.0	military	0.03	plus	0.0	brown	0.05	workers	0.02	is	0.02	at	0.05	hole	0.01	near	0.01	each	0.0	glove	0.01

## 3. Experiments

To evaluate the model, we perform a number of experiments that illustrate how it generates entailments for arbitrary linguistic expressions. The first experiment provides a quantitative assessment of how well the model is able to learn from examples of correct inferential transitions between sentences. Specifically, for a set of novel test sentences, we measure the percentage of correct word-level predictions relative to the entailments for these test sentences present in the dataset. The second experiment provides an empirical assessment of the plausibility of entailments generated by the model for a random selection of novel test sentences. Very roughly, human subjects are asked to rate the likelihood that model-generated entailments are true given that the sentences provided as inputs to the model are also assumed to be true. Together, these two experiments provide an initial quantitative measure of how well the model is able to generate sentences that are the inferential consequences of its inputs.

The remaining assessments of the model expand on these initial measures. The third experiment, following Kolesnyk et al. (2016), involves iterating the encoding-decoding procedure to generate chains of entailments from a given input sentence. Interestingly, this sort of iteration can be used to explicitly build out inferential roles for arbitrary input sentences, as illustrated in section 3.3 below. The fourth experiment involves substituting individual words in an input sentence to identify whether the model is able to “interpolate” between known examples of correct inferential transitions to produce novel transitions that are nonetheless correct. A further goal of this substitutional analysis is to evaluate the extent to which the model is able to learn word-level indirect inferential roles of the sort discussed by Brandom (1994). To measure the sensitivity of the model's predictions to individual words, we collect human plausibility ratings for model generated entailments that are produced by substituting nouns into random collections of input sentences from the SNLI test set. These ratings indicate the degree to which the model is able to generate appropriate entailments from a range of sentences that all contain a specific word. The final experiment is the most speculative in nature, and is designed to condition the model's generation of an entailment on a further input such as a prompt or a question. The goal of this experiment is to evaluate the extent to which the model is able to selectively navigate the inferential roles it assigns to particular sentences. If successful, this kind of selective navigation provides a foundation for more complicated forms of question-answering that many researchers take to be at the core of intelligence (Weston et al., 2015, 2016).

### 3.1. Evaluating entailment accuracy

Within the SNLI corpus, recall, the first sentence in each pair is referred to as the “premise” while the second sentence is referred to as the “hypothesis.” In the procedure just described, the model is essentially learning to predict the hypothesis paired with each example premise in the training data. It is therefore possible to measure how accurately the model performs this task. Specifically, one can measure the proportion of nodes in the model's decoder for which the predicted word is the same as the correct word in the relevant hypothesis sentence. A caveat is that the tree for this sentence must be provided to the decoder, such that input activities are propagated through paired trees of the sort depicted in Figure 2, where the decoder tree is the correct tree for the conclusion of the inferential transition being considered.

When applied to the training set, this accuracy measure indicates the extent to which the model has “memorized” the example inferential transitions it was presented during learning. When applied to the test set, the measure indicates whether the model has learned something that allows it to correctly predict specific inferential transitions in novel situations. It is worth noting that this measure is not entirely ideal in the case of the test set, since the model might generate a plausible entailment from a premise sentence that is non-identical to the specific entailment that is present in SNLI. It is also worth noting that prior work involving SNLI has almost uniformly focused on the problem of classifying sentence pairs. As such, we cannot easily draw comparisons to earlier work, since here we are tackling the more difficult problem of generating a sentence, rather than classifying provided sentences. The literature on “recognizing textual entailment” similarly focuses on classification rather than generation (see e.g., Giampiccolo et al., 2007), and hence is not a suitable target for comparison.

The results of computing entailment generation accuracies on the both training and test sets are presented in Table 3. A baseline accuracy of chance computed via a random initialization of model parameters is also reported. The model performs considerably better than chance, both because it has a large number of free parameters and because it is able to use syntactic information to condition its word predictions on part-of-speech information implicit in the structure of a decoding tree. For example, if the tree requires a particular word to be a determiner, then the number of plausible candidate words shrinks drastically, since there are only a handful of determiners in English (e.g., “the,” “a,” etc.). The model also generalizes reasonably well to novel test sentences, with a fairly limited drop in accuracy.^6^ One point to note concerning this generalization is that extremely high accuracies on the test set are not entirely desirable, since they would indicate that the model has learned to exclusively predict a specific inferential transition for each input sentence. However, there are numerous examples of correct inferential transitions involving such sentences, and the model should ideally be learning to assign a high likelihood to all of them.

**Table 3:** Word-level accuracy for entailment generation.

**Model**	**Training set (%)**	**Test set (%)**
Chance	6.0	5.9
Encoder-Decoder	70.5	60.1

Overall, the fact the model can generate the example inferential transitions in the SNLI test set with a fairly high degree of accuracy provides good initial evidence that it is able to capture the inferential roles of certain ordinary linguistic expressions. Examples of the sort listed in Table 1, moreover, suggest that these inferential roles are often comprised of well-formed sentences that a competent speaker of English could readily understand.

### 3.2. Evaluating entailment plausibility

One limitation of the assessments just described is that they do not provide a quantitative measure of how plausible or comprehensible the sentences produced by the model are. We therefore perform a simple study in which human subjects are asked to evaluate the plausibility of model-generated sentences. During the study, participants are shown a series of sentences introduced as true captions of unseen images. For each caption, the participants are shown an alternate caption and asked to evaluate the likelihood that it is also true of the corresponding image. Evaluations are recorded using a five point Likert scale that ranges from “Extremely Unlikely” (1) to “Extremely Likely” (5). The original caption in each case is the first sentence in a pair randomly chosen from the SNLI test set, while the alternate caption is either (a) model-generated, (b) the SNLI entailment, (c) the SNLI contradiction, or (d) the SNLI neutral hypotheses. An experimental design is used in which participants are all shown the same main captions, but are randomly assigned to see only one of (a-d) as the alternate caption. This ensures both that each participant rates only one caption per premise sentence (so as to avoid order effects), and that each participant sees a mix of all the alternate caption types (so as to avoid different participants implicitly adopting different rating scales). All model-generated sentences were produced using a decoding tree selected at random from the set of twenty decoding trees that were the most frequently used during training.

Eighty participants from the United States were recruited through Amazon's Mechanical Turk. The main captions were identical across conditions, and each participant was asked to rate 20 caption pairs. Participants were paid $0.80 for their time. Four participants failed to complete the study and did not have their responses included in the results. Repeat participation was blocked by screening Mechanical Turk worker IDs. The study was approved by a University of Waterloo Research Ethics Committee, and all participants provided informed consent prior to participation.

The Likert ratings collected during the study are assessments of the plausibility of the inferential transition from one sentence (the main caption) to another (the alternate caption). The transitions involving sentence pairs drawn directly from SNLI offer a kind of gold standard for both good, bad, and neutral transitions. The results shown in Table 4 indicate that model-generated transitions are rated quite positively, and much closer to the SNLI entailments than to the SNLI contradictions. The SNLI neutral hypotheses are rated slightly higher than the model-generated entailments, but this may be due to the fact that these hypotheses are often very *likely* though not guaranteed to be true given the premise sentence. For example, one of the neutral pairs used in the experiment involves an inference from “Child getting ready to go down a slide” to “The child will go down the slide.” Given these considerations, the study provides preliminary evidence in support of the claim that the model is able to generate sentences that are at the very least quite likely to follow from its input.^7^

**Table 4:** Plausibility ratings for inferential relations.

**Source**	**Status**	**Mean likert rating (1–5)**	Confidence interval^*^
Human	Entailment	4.38	[4.31, 4.47]
Model	Entailment	3.59	[3.45, 3.73]
Human	Neutral	3.71	[3.61, 3.81]
Human	Contradiction	1.51	[1.42, 1.60]

* *Margins are bootstrapped 95% confidence intervals*.

To provide a statistical measure of the difference between model-generated entailments and SNLI entailments, we compute Cohen's *d* as measure of effect size. This measure indicates the degree to which an experimental manipulation (e.g., shifting from human-generated to model-generated sentences) alters the distribution of responses. For a comparison of model-generated and human-generated entailments, *d* = −0.703, which indicates that roughly 76% of responses to “Model-Entailment” items are below the mean response for “Human–Entailment” items (Becker, 2000). For a comparison of model-generated entailments and human-generated neutral hypotheses, *d* = −0.101, which indicates that roughly 54% of responses to “Model-Entailment” items are below the mean response for “Human–Neutral” items (Becker, 2000). Given that 50% of responses would be below the mean if these distributions were identical, these quantitative results support our conclusion that the model is able to generate sentences that are at the very least quite likely to follow from its input.

### 3.3. Iteration analysis

**Figure 3:**
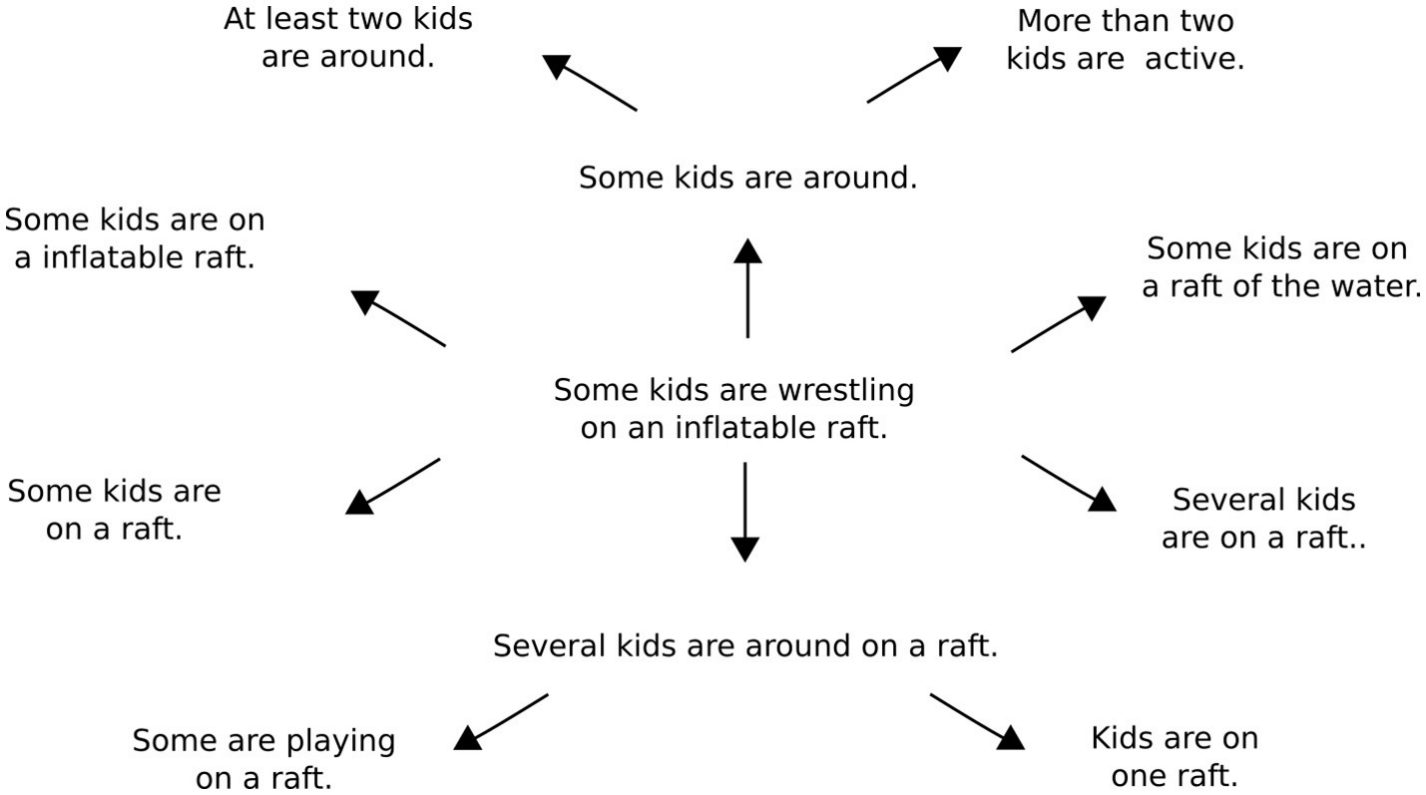
A model-generated inferential network around the sentence “Some kids are wrestling on an inflatable raft.” Each inferential transition is the result of generating a predicted entailment after encoding the sentence at the beginning of each arrow. The entire network is generated starting with only the initial sentence at the center of the diagram, which is drawn from the SNLI test set. Different decoding trees are used to generate the different entailments from the initial sentence.

**Figure 4:**
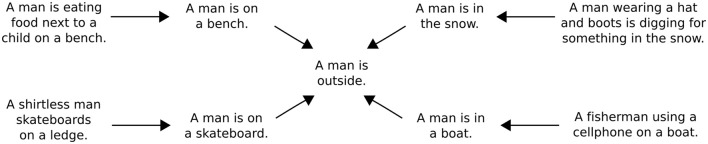
A model-generated inferential network around the sentence “A man is outside.” Each inferential transition is the result of generating a predicted entailment after encoding the sentence at the beginning of each arrow. The entire network is generated starting with only the four outermost sentences, which are drawn from the SNLI test set.

Once an input sentence has been passed through the model to generate an entailment, it is possible to use this entailment as a new input to the model. Repeated applications of the model accordingly make it possible to chart out the inferential role of particular starting sentence. Figure 3 presents a simple example of an inferential role in which the sentence “Some kids are wrestling on an inflatable raft” is mapped onto a number of its inferential consequences. Figure 4 presents a slightly different example in which various sentences describing men doing things outdoors are eventually mapped onto the sentence “A man is outside.” One advantage of using tree-structured rather than recurrent networks in the model is that different decoding trees can be applied to a single sentence encoding, allowing for the generation of multiple entailments from the same sentence.

Two general points can be made here. First, iterative applications of the model can be used to either generate sentences that are (a) increasingly specific, or (b) increasingly general (Kolesnyk et al., 2016). If a predicted entailment is longer than the input sentence, then it tends to describe a more specific situation. For instance, the sentence “A bird is in a pond” can be used to generate the sentence “A little bird is outside in a small pond” by using a decoding tree with nodes for two additional adjectives and an additional adverb. If a predicted entailment is shorter than an input sentence, then it tends to describe a more general situation. For instance, the sentence “A little bird is outside in a small pond” can be used to generate the sentence “A bird is outside” by using a simple decoding tree with four nodes.

Second, these capacities for specification and generalization suggest that the inferential transitions codified by the model can be either inductive or deductive in nature. For example, the inference from “A bird is in a pond” to “A little bird is outside in a small pond” is not strictly truth-preserving and therefore inductive. The inference from “A little bird is outside in a small pond” to “A bird is outside,” on the other hand, *is* strictly truth-preserving and therefore deductive. Interestingly, none of these inferences are formal in the sense that they are licensed strictly by the structure of the input sentence. Rather, they are examples of what Sellars (1953) and Brandom (1994) refer to as *material* inferences, or inferences that are licensed solely by a linguistic expression's meaning.

The most important lesson to draw from this examination of iterative prediction is that it illustrates how the model assigns an inferential role to every possible expression that can be formed from the words in its vocabulary. To explain, the model maps each input sentence onto a set of predictions concerning its inferential consequences. The model can then be used to map each sentence in this set to produce further predictions *ad infinitum*. As such, it is possible to use the model to build networks of the sort shown in Figures 3 and 4 for all possible input sentences. These networks, in turn, are explicit representations of the inferential roles the model assigns to particular sentences. Overall, since the model does not change as it is used to create these networks, it is fair to say that it predicts a network of entailments for every sentence that can be produced from the model's vocabulary.

Of course, nothing guarantees that these inferential roles are appropriate for *all* of the sentences in a given language. It would be rather miraculous if a simple model trained on one hundred thousand entailment pairs managed to *always* generate plausible inferential transitions in novel scenarios. There is nonetheless some degree of fit between the inferential roles defined by this model and the inferential roles that govern the use of ordinary language. The goal of model development, then, is to steadily improve this degree of fit.

### 3.4. Substitution analysis

Proponents of inferential approaches to semantics typically characterize the meanings of individual words in terms of their effects on the inferential roles of the sentences in which they occur (Block, 1986; Brandom, 1994, 2000). The “indirect” inferential role associated with a particular word is then analyzed by swapping it into and out of a variety of different sentences to observe the resulting changes to the kinds of inferences that are licensed by these sentences (Brandom, 1994). Interestingly, the model introduced here can be used to perform this kind of analysis. If individual words in the model's input sentence are replaced, it becomes possible to identify the impact these words have on the inferential transitions that the model predicts. In **Table 6**, for instance, the replacement of a subject noun or a main verb in an input sentence can be seen to have significant effects on the kinds of entailments that are generated by the model.

There are two ways to think about the significance of this substitutional manipulation of the model's behavior. On the one hand, substitution can be used to assess how well the model is able to “interpolate” between the example inferential transitions it was trained on. To explain, any two sentences in the training data can be treated as substitutional variants of one another, provided that enough substitutions are made.^8^ For example, the sentence “The dog chased after the cat” is a substitutional variant of “The woman drove the car”—“dog” is swapped for “woman,” “chased” is swapped for “drove,” “after” is swapped for the empty string, and “cat” is swapped for “car.” If both of these sentences are part of inferential transitions found in the training data, then it is possible to evaluate how the model generalizes beyond these transitions by testing it on inputs that are the substitutional intermediaries of the original sentences. On the other hand, substitutions can also be used to identify specific inferential patterns that are associated with particular expression types (e.g., pronouns, quantifiers, etc.).

To provide an assessment of how well the model learns to accommodate the indirect inferential patterns associated with particular words, we performed an additional experiment in which subjects provide ratings of entailments generated from collections of test sentences that have been modified to include a specific noun. To construct the input sentences used as main captions in the experiment, the following procedure was used. First, ten of the twenty most commonly occurring nouns in the training data were selected at random. Next, for each of these target nouns, twenty premise sentences were randomly chosen from the SNLI test set, and the target noun is used to replace the first occurrence of a noun in each sentence. After a set of twenty sentences per noun is created in this way, we screen each set for semantic anomalies by hand^9^ to produce a set of four novel input sentences involving each target noun. For each input sentence corresponding to a target noun, a model-generated entailment is produced using a randomly selected decoding tree, as before.

Sixty participants from the United States were recruited through Amazon's Mechanical Turk and paid $0.50 for rating ten items. The same experimental design was used, with captions being randomly alternated between entailments generated by the model, entailments from the SNLI test set, and contradictions from the SNLI test set. The inclusion of these entailments and contradictions from SNLI was done to ensure that participant's ratings of the generated subsitutional inferences of interest were appropriately calibrated in relation to clear examples of entailment and contradiction. Five participants failed to complete the study and did not have their responses included in the results.

The results reported in Table 5 indicate that entailments generated from substitutionally-derived input sentences are rated as more similar to clear examples of entailment than to clear examples of contradiction. However, these substitutional entailments are rated somewhat more poorly than the basic model-generated entailments used in the previous study. And while comparisons across experiments are hazardous, it seems reasonable to infer that participants here are using the same rating scale as before, given that their ratings of SNLI entailments and contradictions closely replicate the earlier results. To provide a statistical measure of the difference between model-generated entailments and SNLI entailments in this experiment, we again compute Cohen's *d*. For a comparison of model-generated and human-generated entailments, *d* = −0.949, which indicates that roughly 83% of responses to “Model-Entailment” items are below the mean response for “Human–Entailment” items (Becker, 2000). In all, these results suggest that while the model is not able to always produce accurate entailments on the basis of the inclusion of a specific word in a sentence, the use of word-level substitutions does not drastically reduce the model's ability to generate plausible sentences (c.f. *d* = −0.703 above).

**Table 5:** Plausibility ratings for substitution inferences.

**Source**	**Status**	**Mean likert rating (1–5)**	Confidence interval^*^
Human	Entailment	4.37	[4.21, 4.53]
Model	Entailment	3.26	[3.11, 3.41]
Human	Contradiction	1.46	[1.32, 1.63]

* *Margins are bootstrapped 95% confidence intervals*.

On a more theoretical level, the main benefit of identifying indirect inferential roles is that many of the phenomena that semanticists have traditionally analyzed in truth-conditional terms can be re-analyzed in inferential terms. For example, one can test whether the model generates appropriate entailments for input sentences involving standard quantifiers like “some” and “every.” Similarly, one can test whether the model generates appropriate entailments for input sentences that exhibit anaphoric relations involving pronouns that vary with respect to gender and plurality (e.g., “he” vs. “she” vs. “they,” etc.). Further tests involving expressions that vary with respect to numerals (e.g., one, two, many, etc.) are also possible. It is not reasonable to expect the model to pass all of these tests, since there are relatively few examples of inferential transitions in SNLI that are directly driven by quantification, anaphora, or numerosity. Nonetheless, the model exhibits some promising behavior with respect to these expression types.

In the case of quantifiers, the model is able to infer that “some” and “many” require nouns within their scope to take the plural form in an entailed sentence, as shown in **Table 7**. The model is also able to infer that “some kids” entails “at least two kids,” as shown in Figure 3. In the case of pronouns, the model is sensitive to cues that determine the gender of a pronoun in relation to its anaphoric antecedent. For example, the model correctly infers that girls and women should be referred to with female pronouns, while boys and men should be referred to with male pronouns, as shown in Table 6. In the case of numerals, the model exhibits an ability to infer appropriate quantities from simple groupings and conjunctions. For instance, the model generates a sentence containing the phrase “Two children…” from a sentence containing the phrase “A boy and a girl…” in Table 7. Finally, the model appears to have difficulty with negations. In Table 7, for example, the model incorrectly infers “A boy is not indoors” from “A boy is in a store.” While these results are rather limited, it is worth emphasizing again that the model was not designed or trained to account for phenomena involving quantifiers, pronouns, and numerals specifically. So the fact that the model's predictions are appropriately sensitive to these expressions in some cases suggests that it provides a solid foundation for developing more sophisticated analyses of specific linguistic constructions.

**Table 6:** Substitution analysis for “A boy in a beige shirt is sleeping in a car.”

**Input sentence**	**Generated entailment**
A boy in a beige shirt is sleeping in a car.	A boy is sleeping in his car.
A girl in a beige shirt is sleeping in a car.	A girl is sleeping in her car.
A man in a beige shirt is sleeping in a car.	A man is sleeping in his car.
A woman in a beige shirt is sleeping in a car.	A woman is sleeping in her car.
A man in a beige shirt is driving in a car.	A man is driving a car.
A person in a beige shirt is selling a car.	A person is selling a car.

*Underlining indicates a substituted word*.

**Table 7:** Substitution analysis with quantifiers, numerals, and negations.

**Input sentence**	**Generated entailment**
Some men in red shirts are waiting in a store.	The men are in a store.
Many women in red shirts are waiting in a store.	The women are in a store.
A boy and a girl are waiting inside a store.	Two children are inside.
A boy and a girl are waiting inside a park.	Two children are outside.
A boy is in a car.	A boy is not outside.
A boy is in a store.	A boy is not indoors.

Overall, the extent to which this sort of substitutional analysis can be used to characterize the meanings of individual words is an open question. Words are typically only used in the context of sentences, and sentences, we have argued, have meanings insofar as they license certain inferences. It is accordingly plausible that words have meanings insofar as they help determine which inferences are licensed by the sentences they occur in. Strictly speaking, we endorse this line of reasoning, but it can be misleading if one only considers inferences that relate linguistic expressions to one another, to the exclusion of inferences that relate linguistic expressions to non-linguistic perceptions and actions. In the case of a word like “crayon,” for instance, it would be inadequate to postulate a meaning that merely codifies inferential relations amongst crayon-related sentences while saying nothing about how people identify and use crayons. However, if one could identify all that follows from something being a crayon (both linguistically and non-linguistically speaking), it is difficult to contend that one does not know what the word “crayon” means.

### 3.5. Conditioned entailments

Up to this point, the association of particular inferential roles with particular sentences has not lead to any concrete explanations of facts concerning the *use* of these sentences. To build toward such explanations, we briefly examine various methods for conditioning the model's predictions on additional inputs. The idea is to selectively navigate the inferential role associated with a particular sentence so as to provide appropriate answers to specific questions about the sentence. To illustrate with a hypothetical example, consider once more the sentence “The dancers parade down the street.” Providing an answer to a question such as “Are the dancers outside?” involves drawing one inference amongst the many that are licensed by the original sentence. More generally, every answer to a question about this particular sentence is simply a different sentence specified by its inferential role.

There are two reasons why question answering is worth exploring. First, the matter of whether a model can adequately perform simple forms of question answering is highly relevant to determining whether or not its behavior can be predicted by adopting the intentional stance. Put simply, a system that *understands* a particular linguistic expression will undoubtedly be able to answer certain questions about it (St. John and McClelland, 1990; Rabovsky et al., in review). Given our supposition that the expectations set out by inferential roles are what make intentional interpretation possible,^10^ it is important to verify that the model can be subjected to such interpretation. Second, an examination of question answering allows for a clear connection to be drawn between the inferential roles assigned to particular expressions and the *use* of those expressions. For example, the assignment of an inferential role to a sentence helps to explain, amongst other things, how it gets used in simple question-and-answer dialogues.

As an initial test of the model's ability to generate conditioned entailments, we supplement its input with simple prompts consisting of single words. The resulting change to the encoding procedure is quite minimal. First, an input sentence is converted into an embedding using the usual tree-structured encoder. Second, a word embedding corresponding to a prompt is added to this embedding. The resulting sum is then passed through the decoder to produce a predicted entailment. The effect of this process is to subtly shift the input sentence embedding toward the prompt embedding, with the expectation that this shift will be reflected in the prediction of an entailment that is appropriate to the prompt. Table 8 illustrates some examples of the kinds of the entailments that the model predicts under these conditions.

**Table 8:** Prompts with “A man is steering his ship out at sea.”

**Prompt**	**Generated entailment**
Water	A man is in the water.
Fish	A man fishes in the water.
Sails	A boat sails in the sea.
Steering	A man steering in the water.
Voyage	A ship sailing in the sea.
Sea	A sea sea in the sea.

The natural next step is to use complete questions instead of single word prompts to condition the model's predictions. To take this next step, we modify the encoding procedure to produce *two* sentence embeddings using two separate encoding trees. The first embedding corresponds to an input sentence, while second embedding corresponds to a question. These embeddings are then added together before being passed to the decoder network. The idea, again, is that shifting the input embedding toward the question embedding will force the decoder to predict an entailment that is an answer to the question. An important caveat is that the model was not trained to perform this task, so there is little reason to suppose that it will produce appropriate answers. As Table 9 indicates, the answers the model provides in response to questions are often not particularly illuminating. Nonetheless, these answers generally provide relevant information for the question provided.

**Table 9:** Queries with “A mother and daughter walk along the side of a bridge.”

**Query**	**Generated entailment**
How many people are walking?	Two people are walking.
What are the people doing?	A people are together on a water.
Where are the people?	The people are on a water.
How fast are the people walking?	A people walking very close.
What is the bridge over?	The people is on a bridge.

Tables 9 and 10 together illustrate that it is possible to qualitatively examine the relative importance of queries and input sentences. For example, if the input sentence is altered while the queries are held constant,^11^ it is possible to isolate the changes in the predicted answers that are due to properties of the input sentence specifically (i.e., the queries and the decoding trees are held constant). As is illustrated in these tables, the inclusion of the word “bridge” in the input sentence seems to help surface answers that highlight the proximity of water, while the inclusion of the word “street” seems to help surface answers that highlight being outside. Varying the queries further could help to determine the range of sensitivity that generated entailments have given a fixed input sentence and decoding tree. It may be, however, that some of the observed variation is due to the decoding tree rather than the query *per se*, and as such, it is not yet entirely clear how inputs, queries, and the decoding structure interact to produce a predicted entailment.^12^

**Table 10:** The same queries with “A mother and daughter walk along the street.”

**Query**	**Generated entailment**
How many people are walking?	Two people are walking.
What are the people doing?	A people are outside with the street.
Where are the people?	The people are on the street.
How fast are the people walking?	A people walking very present.
What is the street over?	The people are down the street.

Overall, these tests are merely suggestive, but they point toward the development of more sophisticated models for which performance on conditional inference tasks is incorporated directly into the training objective. Developing such models will undoubtedly require training data comprised of numerous example question-answer pairs for each input sentence of interest. There are currently a number of engineering-driven efforts to build systems that learn to answer questions about short collections of text (e.g., Sukhbataar et al., 2015; Weston et al., 2015, 2016), but these efforts have not lead to the creation of publicly available datasets of the required sort.

## 4. Discussion

The primary purpose of this work is not to advance the technical state-of-the-art in neural network modeling. Rather, its purpose is to illustrate how neural networks can be used to formalize a particular approach to thinking about the meaning of language. This approach, again, involves treating linguistic expressions as instruments of prediction that play a role in social practices involving intentional interpretation. The meaning of a linguistic expression, then, can be specified in terms of the predictions and inferences it licenses in the context of intentional interpretation. A key theoretical shift that results from this way of thinking is that the meanings of linguistic expressions should be characterized primarily in terms of their inferential relations to one another (along with certain non-linguistic perceptions and actions), rather than primarily in terms of the properties of underlying representations. Or put another way, the job of characterizing an expression's meaning involves specifying the inferential relations that it gets caught up in rather than specifying the features of particular mental representations that get associated with particular words and sentences. One of our main goals has been to argue that neural networks are a promising tool for carrying out this job (e.g., by allowing one to automatically generate networks like those in Figures 3 and 4).

This inferentialist approach to semantics is related to a number of strands of earlier research. On a technical level, the idea of using neural networks to learn relations amongst sentences has been developed in a body of work on modeling story comprehension with RNNs (Golden and Rumelhart, 1993; Frank et al., 2003, 2009). However, our encoder-decoder model expands on this prior work in three important ways. First, there is no hand coding of linguistic expressions or the constraints that hold between them; everything is learned automatically from real-world language data. Second, the model scales to a realistic vocabulary size and a realistic range of sentence types with sophisticated syntactic structures. Third, and perhaps most importantly, we incorporate language generation into our modeling framework.

On a more theoretical level, the inferentialist approach is closely related to work on procedural semantics (Johnson-Laird, 1977) and natural logic (Lakoff, 1970). In the case of procedural semantics, our emphasis on processes of inference rather than representational states is clearly in line with the proceduralists' call to consider “processes as well as structures” in the development of a psychologically plausible semantic theory (Johnson-Laird, 1977, p. 193). Our approach differs, however, in both (a) characterizing the relevant processes in terms to a theory of how linguistic expressions are used as instruments of prediction in context of social interaction, and (b) avoiding the assumption that comprehension involves building up a representational structure that constitutes a “semantic interpretation” of an input sentence (p. 195).^13^ In the case of natural logic, our work shares an emphasis on producing entailments without appeal to logical forms that deviate from a sentence's grammatical form. On the other hand, a significant difference is that we do not introduce explicit inference rules that can be used to produce a step-by-step derivation of an entailed sentence from a starting sentence. Further work could profitably explore the relationship between our inferentialist approach and natural logic in more detail.

More broadly, if the inferentialist approach is on the right track, then there are some important implications for the study of the cognitive mechanisms that underlie language use. Again, if understanding a linguistic expression involves forming certain predictions and drawing certain inferences, then it is reasonable to shift from thinking about representational *states* that encode sentence meanings as structured objects to thinking about inferential *processes* that determine the roles sentences play in an individual's behavioral economy. Two areas in which this shift is of particular importance include (a) debates about the principle of compositionality (Szabo, 2013), and (b) debates about the role of syntax in language processing.

With respect to (a), our process-based approach to thinking about language use and linguistic cognition is incompatible with standard formulations of the principle of compositionality, on which complex “meanings” are built up out of simpler ones (Fodor and Lepore, 1991; Szabo, 2013). The approach is, however, compatible with a procedural notion of compositionality on which certain procedures get re-used when determining the inferential consequences of novel linguistic expressions (Blouw, 2017), as in our model. Notice too that the main motivation for postulating the principle of compositionality is to explain how people are able to generalize from the use of familiar linguistic expressions to the use of unfamiliar ones (Fodor and Pylyshyn, 1988; Fodor and Lepore, 2002; Szabo, 2012). But if so, then debates about the principle are really about generalization rather than semantic composition *per se*. Generalization, in turn, can be achieved in many different ways, and it is not entirely clear that language users generalize on the basis of structural rules of the sort typically proposed by linguists (Tomasello, 2003). Moreover, it is plausible that one way in which language users generalize is by “interpolating” between familiar examples of good inferential transitions, as illustrated in section 3.5.

With respect to (b), it is worth noting that our approach fits well with the idea that a sentence's syntactic structure is akin to description of its processing history (Lupyan and Clark, 2015; Christiansen and Chater, 2016). The encoder-decoder model, to explain, never constructs explicit syntactic representations of its inputs. Rather, the role of syntax in the model is to guide the procedure by which word embeddings are transformed into sentence embeddings, and vice versa. None of these embeddings possess explicit constituent structure; a sentence embedding, for instance, is not a syntactically structured “whole” that is comprised of “parts” corresponding to individual word embeddings (Eliasmith, 2013). An interesting consequence of this observation is that embeddings cannot be manipulated by purely formal inference rules, since such rules, by definition, operate on structures comprised of parts and wholes (Fodor and Pylyshyn, 1988; Marcus, 1998).

## 5. Conclusion

In summary, the point of this work is to motivate an approach to semantics based on inferential relationships (Brandom, 1994). The use of the encoder-decoder model is designed to illustrate how very simple inferential roles can be learned for arbitrary linguistic expressions from examples of how sentences are distributed as tacit “premises” and “conclusions” in a space of inferences. It is accordingly possible to characterize this work as an extension to the well-known distributional approach to semantics (Turney and Pantel, 2010), wherein the generic notion of a linguistic context is replaced with the more fine-grained notion of an inferential context.

As with most natural language generation systems, many of the sentences produced by the model are defective in some way. As can be seen in the examples in Tables 8 and 9, model-generated entailments are almost always thematically appropriate, but sometimes contain agreement errors or misplaced words that render the entailment as a whole ill-formed. And, not infrequently, the model produces entailments that are more or less incomprehensible. There are two ways to address these problems. The first involves the use of increased amounts of training data to provide the model with a more points in the “space of inferences” to interpolate between. The second involves the use of more sophisticated network architectures that help the model to learn to more selectively make use of only the input information that is most relevant to generating a good entailment. Tree-structured architectures such as the Tree LSTM (Tai et al., 2015; Zhu et al., 2015), the Recursive Neural Tensor Network (Socher et al., 2013), or the lifted matrix-space model (Chung and Bowman, 2017) can potentially provide improvements on this second front.

Finally, an important limitation of this work is that it does not directly consider the relationship between linguistic expressions and the non-linguistic world. A natural way to account for this relationship is to suppose that a sentence's occurrence in the linguistic environment licenses certain expectations about what can be seen, heard, or otherwise perceived. To return to our initial example, if one understands the statement “The dancers parade down the street,” one will expect to see and hear dancers upon going to the relevant street. We accordingly suggest that if an individual can adequately infer all that follows from a given linguistic expression, both linguistically and non-linguistically, then there is nothing further they need to be able to do to count as *understanding* what the expression means.

## Code and data

All of the experiments described in this paper were implemented using a neural network library written by the first author, available at https://github.com/pblouw/pysem. Code for running simulations, along with data from the studies described in Sections 3.2 and 3.4, is available at https://github.com/pblouw/frontiers2018.

## Author contributions

PB designed the study, wrote the code, carried out the experiments and analyzed the data. PB wrote the manuscript. CE contributed to the conception of the study and helped draft the manuscript.

### Conflict of interest statement

The authors declare that the research was conducted in the absence of any commercial or financial relationships that could be construed as a potential conflict of interest.

## Footnotes

^1^Note that the strategy of tying a theory of semantics to to a theory of intentional interpretation does *not* imply a commitment to the existence of a language of thought, or to the psychological reality of intentional states involving beliefs and desires. Note also that recent machine learning efforts related to natural language understanding (or NLU) seem to implicitly make use of the intentional stance when they operationalize understanding in terms of providing expected answers to particular questions (see e.g., Sukhbataar et al., 2015; Weston et al., 2015, 2016).

^2^We use the SpaCy python library, available at https://spacy.io

^3^These annotators were recruited through Amazon Mechanical Turk. See Bowman et al. (2015) for details.

^4^We use the PyEnchant python library, available at http://pythonhosted.org/pyenchant/.

^5^Note that the probabilities listed in Table 2 are rounded to two decimals, and so may not sum exactly to 1 in all cases.

^6^If the model were merely memorizing the example inferential transitions present in the training data, then this drop would likely be much higher.

^7^Notice too that if a selected decoding tree requires producing an entailment that is *longer* than the input sentence, then it is likely that new information will have to be added that will prevent the predicted inferential transition from being strictly truth-preserving (simply because more words are now present). Thus, anytime the generated sentence is longer than the input sentence, the Neutral category of sentence pairs might be the appropriate target of comparison.

^8^We include insertions and deletions, which can be thought of as substitutions involving the empty string.

^9^Simply replacing one noun in a sentence with another often yields infelicitous results. For example: “Two girls wearing hats are running through person drifts outside.”

^10^The idea, recall, is that intentional state attributions only license certain behavioral predictions because the sentences invoked by these attributions have particular inferential roles.

^11^Barring the changes that are required to ensure the questions remain applicable to the altered sentence. For example, the question “What is the bridge over?” is changed to “What is the street over?” when the input sentence is changed from “A mother and daughter walk along the side of a bridge” to “A mother and daughter walk along the street.”

^12^Note that it is not possible to use the same decoding tree in all cases, since it typically not possible to produce appropriate answers to different questions using sentences that all share an identical syntax.

^13^This latter assumption is actually somewhat odd, since it is in tension with the claim that procedural semantics is actually an alternative to common denotational semantics, since a denotation relation of some kind is presumably what permits a representational structure to be interpreted in isolation. See Hadley (1989) for discussion related to this point.
